# IL-33 and the PKA Pathway Regulate ILC2 Populations Expressing IL-9 and ST2

**DOI:** 10.3389/fimmu.2022.787713

**Published:** 2022-05-30

**Authors:** Enrique Olguín-Martínez, Ofelia Muñoz-Paleta, Blanca E. Ruiz-Medina, Jose Luis Ramos-Balderas, Ileana Licona-Limón, Paula Licona-Limón

**Affiliations:** ^1^Departamento de Biología Celular y del Desarrollo, Instituto de Fisiología Celular, Universidad Nacional Autónoma de México, México City, Mexico; ^2^Immunobiology Department, Yale University, New Haven, CT, United States

**Keywords:** IL-9, ILC2, ST2, IL-33, PKA, pathway, regulation

## Abstract

Type 2 Innate lymphoid cells (ILC2s) are tissue-resident immune cells activated by epithelial-derived alarmins upon tissue damage. They regulate immunity against helminth parasites and allergies by expressing type 2 immune response cytokines including IL-9, known to be critical for inducing and potentiating the immune response in such context. Although ILC2s are reported to be the main source of IL-9 in mice during *N. brasiliensis* infection, the mechanisms that regulate the expression of IL-9 in these cells are yet to be described. Recent studies have shown that in addition to cytokines, multiple molecules can differentially modulate the functions of ILC2s in various contexts both *in vitro* and *in vivo*. Among these stimuli are lipid mediators and neuropeptides, which activate the PKA pathway and have been associated with the regulation of type 2 immune cytokines. In this work we found that ILC2s in mice infected with *N. brasiliensis* can be classified into different groups based on the expression of IL-9 and ST2. These distinct populations were distributed in the lung and the small intestine. Through the development of an *in vitro* culture system, we sought to determine the stimuli that regulate the expression of these markers in ILC2s. We identified the alarmin IL-33 as being a key player for increased IL-9 expression. Additionally, we found the PKA pathway to be a dual regulator of ILC2 cells, working synergistically with IL-33 to enhance IL-9 production and capable of modulating proliferation and the expression of ILC2 markers. These data provide further evidence of a high heterogeneity between ILC2 subsets in a context dependent manner and calls for careful consideration when choosing the markers to identify these cells *in vivo*. Distinguishing ILC2 subsets and dissecting their mechanisms of activation is critical for a deeper understanding of the biology of these cells, allowing their manipulation for therapeutic purposes.

## Introduction

Innate lymphoid cells (ILC) are tissue-resident immune cells lacking receptors to recognize specific antigens; hence their activation depends on cytokines present in the microenvironment (1). Type 2 ILCs or ILC2s are mainly found in the lung, intestine and skin, where they are activated by the epithelial cell-derived alarmins IL-25, TSLP, and IL-33 upon tissue damage (2), although they can also respond to cytokines derived from the immune compartment such as IL-2 and IL-7 (3).

ILC2s are mainly tissue resident cells; however, their precursors reside in the bone marrow, where they maturate after going through different stages of differentiation. Migration of ILC2 precursors from the bone marrow to different peripheral tissues such as skin and lung has been shown to occur in response to intravenous IL-33 administration or IL-33-dependent pulmonary fungal allergen challenge (4). In addition, intranasally administered IL-33 can have a direct effect on bone marrow derived ILC2s, inducing their production of IL-5, demonstrating an important role for these cells in the induction of eosinophilia derived from inflammation in the upper airways (5).

Recent studies have shown that multiple molecules besides cytokines can regulate the functions of ILC2s in different contexts both *in vitro* and *in vivo*. These stimuli include the lipid mediators leukotrienes and prostaglandins, neuropeptides, hormones and nutrients (6), thereby demonstrating that the regulation of ILC2 function is tightly controlled by a variety of signals within the cellular microenvironment. Several of these stimuli act through the cAMP/PKA pathway.

Cyclic adenosine monophosphate (cAMP) has been established as a universal regulator of metabolism and gene expression in all living organisms (7). The levels of this messenger are regulated by different enzymes such as adenylate cyclase and phosphodiesterases (8). Although cAMP acts in multiple downstream pathways, its most prominent role is the activation of the protein kinase A (PKA). Upon binding of cAMP, PKA dissociates into its regulatory and catalytic subunits. The catalytic subunits then phosphorylate specific Ser and Thr residues on numerous downstream target proteins involved in different signaling pathways. For example, cAMP-activated PKA binds and phosphorylates cAMP-responsive transcription factors, including cAMP-response element binding protein (CREB), activating transcription factor-1 (ATF-1), Nuclear Factor κB (NFκB), and nuclear receptors (8).

Studies exploring the effects of cAMP, PKA or CREB activation on ILCs are limited. However, on helper T cells, ILCs adaptive cellular counterparts, they regulate cytokine expression, proliferation and apoptosis (8–12). Altogether these reports suggest that activation of the cAMP/PKA pathway has a negative effect on T cell survival by inhibiting cell proliferation and increasing apoptosis (10–12). The cAMP pathway was recently shown to suppress ILC2 function through mediators such as PGE2 and PGI2 (13, 14). Contrastingly, cAMP signaling mediated by neuropeptides activates ILC2s (6), demonstrating a dual regulation of these cells that is context dependent.

ILC2s are characterized by the expression of cytokines associated with the type 2 immune response, including IL-4, IL-5, IL-13 and IL-9, all important in the regulation of immunity against helminth parasites and allergies (15). The expression of IL-9 derived from ILCs was first confirmed in 2011 using a fate reporter in models of sterile inflammation of upper airways (16). A couple of years later, the first evidence of IL-9 expression specifically in ILC2s *in vivo* was reported, when ILC2s were found to be the main source of IL-9 expression in mice during *Nippostrongylus brasiliensis (N. brasiliensis)* infection (17). ILC2 cells were confirmed to express high levels of the IL-9 receptor (18) and this cytokine was found to act in an autocrine manner, promoting IL-5, IL-13 and amphiregulin expression as well as ILC2 survival (19). Despite these findings, the mechanisms controlling the expression of IL-9 in ILC2s remain unclear.

Most studies on the regulation of ILC2 cytokines by cAMP-associated pathways have been focused on IL-5 and IL-13 in different inflammation models (13, 20). However, studies exploring how the cAMP pathway could potentially regulate IL-9, and cellular processes such as proliferation and survival, are lacking.

Suppressor of Tumorigenicity 2 (ST2), a component of the IL-33 receptor, is commonly used as a marker for the identification of ILC2s. In mice, ST2 is expressed on ILC2s in the lung, and can also be found in bone marrow and adipose tissue, while its basal expression is limited in tissues such as the gut and skin (21, 22). IL-33 signals through ST2 to activate ILC2s, which produce IL-5 and IL-13 in models of upper airway inflammation and helminth infection (23–25).

In this work, we explored the signals that control IL-9 expression in ILC2s and how the regulation of PKA directs the function of these cells. We found that during *N. brasiliensis* infection, different populations of ILC2s can be distinguished based on their IL-9 and ST2 expression. These populations are distributed in the different anatomical compartments analyzed. *In vitro*, IL-33 and the cAMP/PKA pathway modulated the proliferation of ILC2s and regulated their expression of IL-9 and other markers, establishing the stimuli that could be regulating the heterogeneity and function of these cells in the different compartments during infection with *N. brasiliensis in vivo*.

## Experimental Procedures

### Mice

All mice used were on C57BL/6 background at 8–12 weeks of age. INFER and KN2 mice were generated and genotyped as previously described, used in heterozygosity (17, 26) and maintained according to the bioethics and biosecurity norms from the Instituto de Fisiología Celular in the National University of Mexico.

### *N. brasiliensis* Infection

Mice were infected subcutaneously with 200 viable third stage *N. brasiliensis* larvae. Animals were sacrificed at different time points post-infection, and tissues harvested and processed for flow cytometry staining as described below.

### Tissue Processing

The mediastinal and mesenteric lymph nodes were isolated, homogenized, filtered and resuspended in RPMI media supplemented with 10% fetal bovine serum (FBS) for subsequent analysis by flow cytometry. The lung was isolated and digested with 1 mg/mL collagenase D (Sigma) and 20 µg/mL DNAse (Sigma). The samples were subsequently homogenized and subjected to an Optiprep gradient followed by resuspension in medium for analysis by flow cytometry. The small intestine was processed according to the protocol of Ferrer-Font et al. (27) with some modifications for the recovery of intraepithelial cells. Cells isolated from the small intestine are extremely sensitive and viability is compromised with prolonged processing times; hence, we recommend working as fast as possible with no more than 3 mice simultaneously. In short, the small intestine was isolated and the Peyer patches were extracted. The intestine was then opened longitudinally, cut into small pieces, and washed 3 times with PBS. The small intestine pieces were incubated with EDTA solution (2mM) in HBSS, and the supernatants were recovered for the analysis of intraepithelial cells. These supernatants were washed once with PBS and resuspended in RPMI media supplemented with 10% FBS and 20 µg/mL DNAse (Sigma) for subsequent staining and analysis by flow cytometry. For lamina propria cell isolation, the remaining tissue was digested with 1 mg/mL collagenase D (Sigma) and 50 µg/mL DNAse (Sigma) for 30 minutes shaking vigorously every 5 minutes. The samples were then passed through a cell strainer, homogenized, washed once with PBS and resuspended in RPMI media supplemented with 10% FBS and 20 µg/mL DNAse (Sigma) for subsequent staining and analysis by flow cytometry.

### *In Vitro* Bone Marrow Cultures

Bone marrow was extracted from femur, tibia and humerus of mice. Cell suspensions were prepared after ACK erythrocyte lysis and resuspended in RPMI medium supplemented with 10% FBS, penicillin (100 Units/mL), streptomycin (100 µg/mL), glutamine (292 µg/mL) and 2-mercaptoethanol (50 μM). Bone marrow cultures were performed in the presence of 10 ng/mL of either IL-2 (Peprotech), IL-7 (Peprotech), TSLP (Peprotech), or IL-25 (eBioscience) with or without 10 ng/mL IL-33 (Peprotech). 100 μM 8-Br-cAMP (Sigma) was added to the cultures where indicated. The cells were incubated at an initial density of 1 x 10^6^ cells/mL in 48-well plates at 37°C in 5% CO_2_. Media was refreshed every 4 days where appropriate.

### *In Vitro* ILC2 Cultures (BM-ILC2 Cultures)

Whole bone marrow cells were cultured for 3 days in the presence of IL-2, IL-7, TSLP and IL-25 at an initial density of 2 x 10^6^ cells/mL under previously specified culture conditions. Sorted ILC2 (CD45+ lineage- CD90^+^ CD25+) were recovered in fetal bovine serum and resuspended in RPMI medium supplemented with 10% FBS, penicillin (100 Units/mL), streptomycin (100 µg/mL), glutamine (292 µg/mL) and 2-mercaptoethanol (50 μM) and containing 10 ng/mL of either IL-2 (Peprotech), IL-7 (Peprotech), TSLP (Peprotech), or IL-25 (eBioscience). 10 ng/mL IL-33 and 100 μM 8-Br-cAMP (Sigma) was added to the cultures where indicated. The cells were incubated at an initial density of 4 x 10^4^ cells/mL in 96-well plates at 37°C in 5% CO_2_ for 4 days.

### Flow Cytometry

For the lineage cocktail, biotin-coupled antibodies against B220, CD4, CD8, CD11b, CD11c, CD19, FcϵRI, Gr-1, NK1.1, TCRβ, TCRγδ, Ter119 and SiglecF (eBioscience) were used. Fluorescent coupled streptavidin or antibodies against CD45, ST2, CD90^+^, human CD2, CD25, KLRG1 and CD127 (Biolegend) were used.

For intracellular staining antibodies against IL-5 (BD Pharmingen), IL-13 (eBioscience) and GATA-3 (eBioscience) were used. To determine the cell viability, we stained the cells using Zombie aqua reagent (Biolegend). The ILC2s were sorted using a Facs ARIA II sorter in the Instituto Nacional de Enfermedades Respiratorias (INER) and the Instituto de Investigaciones Biomédicas, UNAM. The samples were analyzed using an Attune Nxt cytometer (Thermofisher) located in the Laboratorio Nacional de Citometría de Flujo of Instituto de Investigaciones Biomédicas, UNAM and a BD FACSMelody Cell Sorter located in the Instituto de Fisiología Celular.

### Proliferation Assays

Cultures were stained on day 3 of whole bone marrow culture before sorting for ILC2s with the Tag-it Violet reagent according to the manufacturer’s instructions (Biolegend). Proliferation assays were performed by following the dilution of the violet dye by flow cytometry and the division index was calculated using the FlowJo software.

### Apoptosis Determination

Cells were incubated with viability dye Zombie (Biolegend), stained for surface markers and incubated with annexin V (eBioscience) after staining according to the manufacturer instructions. The frequencies of living, apoptotic and necrotic cells were determined by flow cytometry.

### Statistical Analysis

Statistical analyzes were calculated in Prism 6.0 (Graphpad Software) using a two-tailed Student’s test. Experiments with more than two groups, were analyzed using one way and two-way ANOVA tests with *post hoc* Tukey and Bonferroni tests. p ≤ 0.05 was considered significant.

## Results

### Helminth Infection Results in the Generation of Distinct ILC2 Populations

**Figure 1:**
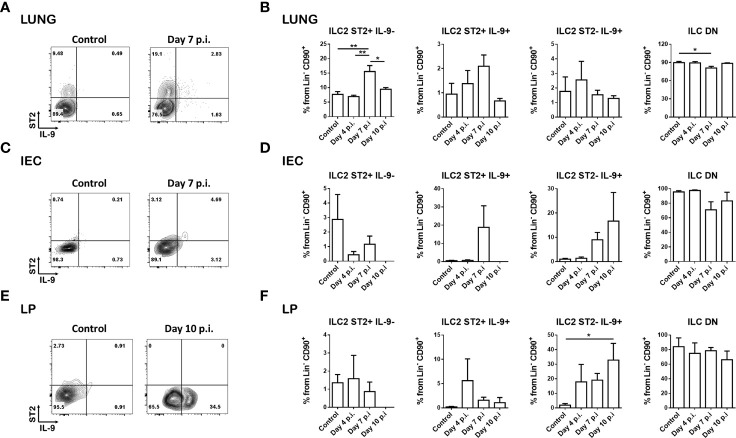
Helminth infection results in distinct ILC2 populations. INFER mice were subcutaneously infected with 200 N*. brasiliensis* larvae and ILC2s from lung and small intestine were analyzed at different times post-infection. **(A)** Representative dot plots of the ILC2 populations generated in the lung on day 7 post-infection compared to day 0 (control) (gated on live CD45^+^ lineage^-^ CD90^+^ cells). **(B)** Frequencies of the ILC2 subsets in the lung on day 0 (control), 4, 7 and 10 post-infection. **(C)** Representative dot plots of the ILC2 populations generated in the intraepithelial cells (IEC) compartment on day 7 post-infection compared to day 0 (control) (gated on live CD45^+^ lineage^-^ CD90^+^ cells). **(D)** Frequencies of the ILC2 subsets in the intraepithelial cell compartment on day 0 (control), 4, 7 and 10 post-infection. **(E)** Representative dot plots of the ILC2 populations generated in the lamina propria (LP) on day 10 post-infection compared to day 0 (control) (gated on live CD45^+^ lineage^-^ CD90^+^ cells). **(F)** Frequencies of the ILC2 subsets in the lamina propria on day 0 (control), 4, 7 and 10 post-infection. Data shown are mean ± SEM. n = 2 for day 4 and day 10 experiments; n = 4 for day 7 experiments; 1-2 mice were analyzed per experiment. *p < 0.05, **p < 0.01.

ST2 is one of the main markers used to specifically identify ILC2s within the ILC group. The presence of this marker on ILC2s has been reported in different organs and study models *in vivo* (23, 28, 29). Along with ST2, expression of type 2 immune response cytokines is also a unique feature of this population. Within these type 2 cytokines, IL-9 plays an essential role in the immune system defense against helminth infections (17). However, this cytokine has only been reported to be present under inflammatory conditions (16, 17). In order to assess the expression of ST2 and IL-9 in ILC2s, we infected IL-9 reporter mice (INFER) with *N. brasiliensis* larvae and analyzed lung and small intestine tissue at 0, 4, 7 and 10 days post-infection. The IL-9 INFER reporter allows tracing of cells that express this cytokine without altering expression levels when used in heterozygosity as previously reported (17). Evaluation of the simultaneous expression of IL-9 and ST2 revealed the presence of separate ILC2 populations specific to the analyzed compartment (**Figure 1**; **Supplementary Figure 1A**).

In the lung at steady state conditions, we found most of the ILC2s to be ST2^+^ and IL-9^-^, with only a small percentage of ILC2s expressing IL-9 (**Figures 1A, B**). At 7 days post-infection, we observed an increase in this predominant ST2^+^ IL-9^-^ population along with trending increases in the double positive ILC2 population, and a slight but significant decrease in the double negative ILC population (**Figures 1A, B**; **Supplementary Figure 1B**).

In the small intestine within the intraepithelial cell compartment, most ILCs are double negative under basal conditions (**Figure 1C**). At day 7 and 10 post-infection, there were trending increases in the ST2^+^IL-9^+^ and ST2^-^IL-9^+^ populations (**Figure 1C**), however these differences were not statistically significant (**Figure 1D**). In the lamina propria, a low expression of ST2 was observed, along with a small percentage of ST2^+^ IL-9^-^ cells that remained unchanged with infection. In contrast, we observed an increase trend in the double positive population at day 4 post infection, and a significant increase in the ST2^-^ IL-9^+^ population on day 10 post infection (**Figures 1E, F**).

Analyses of lung and small intestine draining lymph nodes revealed increased frequencies and numbers of these ILC2 populations on day 10 post infection, mostly in the mesenteric lymph nodes (**Supplementary Figures 1C–F**).

Overall, we observed a clear distribution of ILC2 populations based on the expression of ST2 and IL-9 in the infected tissues. Importantly, the expression of these markers is not conserved among tissues and seems to be regulated in a microenvironment-specific manner.

### Development of a Novel Bone Marrow-Derived ILC2 Culture Model

**Figure 2:**
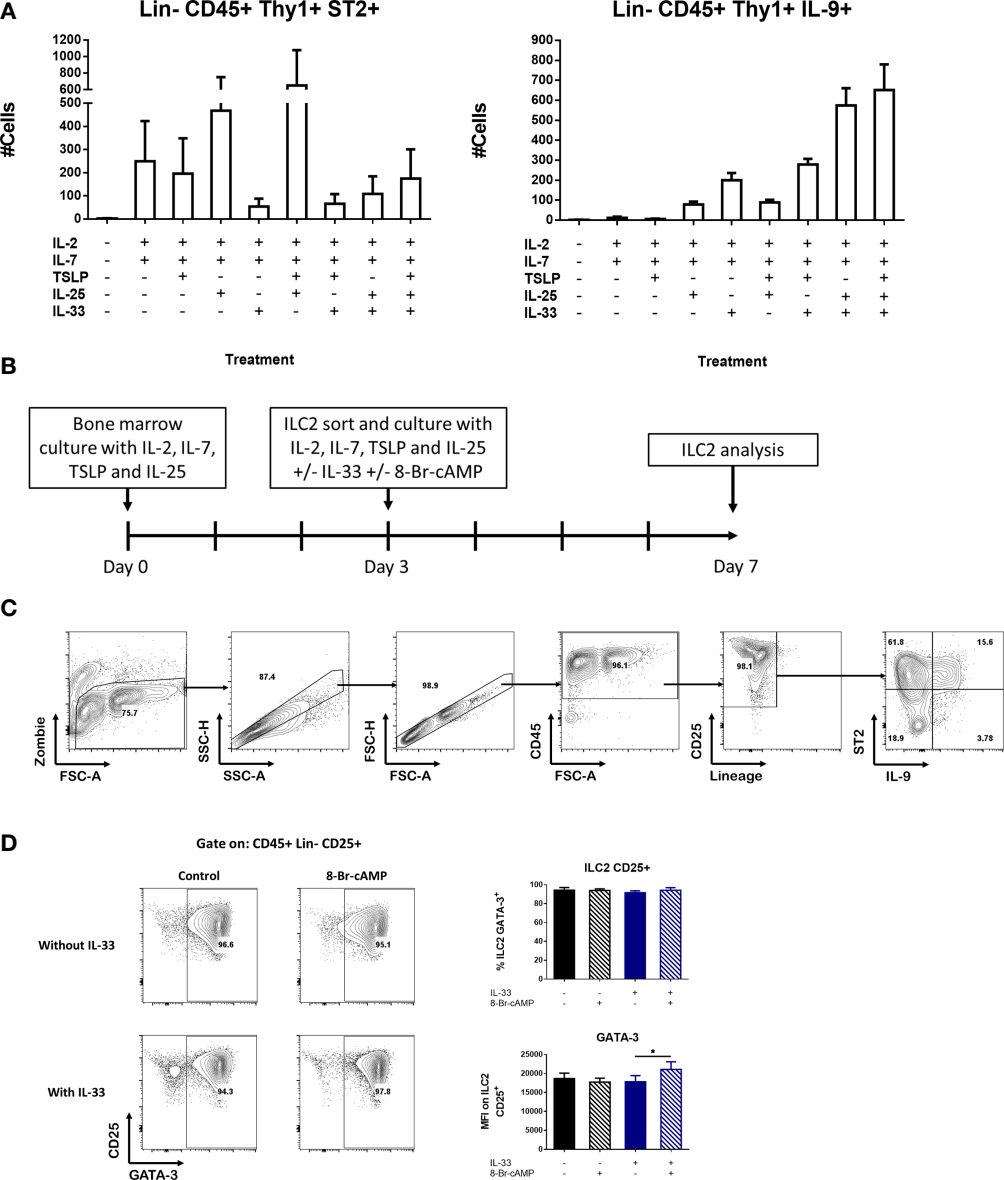
Novel bone marrow-derived ILC2 culture model. **(A)** Induction of ILC2 numbers expressing ST2 or IL-9 in the presence of different combinations of cytokines in whole bone marrow cultures. The medium was changed at day 4 and the cells were analyzed at day 7. Data represent the mean ± SEM of three mice per group. **(B)** Schematic of the protocol used for the BM-ILC2 culture. **(C)** Gating strategy to identify populations in the BM-ILC2 culture. **(D)** Representative dot plots of ILC2 and graphs of GATA-3 expression in BM-ILC2 at day 7. Data represent the mean ± SEM of four mice per group analyzed in four independent experiments. *p < 0.05.

To determine which signals contribute to the generation of different ILC2 populations in tissues, we developed an *in vitro* differentiation model. First, cells extracted from bone marrow were cultured with different combinations of cytokines known to be important for the differentiation and proliferation of ILC2s including IL-2, IL-7, IL-25, TSLP and IL-33 and selected the combinations that resulted in the highest number of ST2 or IL-9 positive ILC2s (**Figure 2A**). We observed that the combination of IL-2, IL-7, TSLP and IL-25 induce the highest numbers of ST2^+^ ILC2s with low expression of IL-9 (**Figure 2A**). IL-33 is known to induce bone marrow ILC2s to produce IL-5 locally and to stimulate their exit towards the periphery (4, 5). Interestingly, adding IL-33 to our culture resulted in increased frequency of IL-9^+^ cells within the lineage^-^ CD90^+^ population (**Figure 2A**).

Based on this information, we designed a protocol to expand ILC2s termed BM-ILC2 culture (**Figure 2B**) in which we performed a 3-day bone marrow culture in the presence of cytokines (IL-2, IL-7, IL-25 and TSLP) that induce ST2^+^ ILC2 cells. After this incubation period, we sorted the ILC2s identified as CD45^+^ lineage^-^ CD90^+^ CD25^+^ (**Supplementary Figure 2A**), and cultured them an additional 4 days with the same cocktail of cytokines in the presence or absence of IL-33. ILC2 cells cultured without IL-33 express ST2 and therefore are capable of responding to this alarmin, hence the addition of IL-33 post-sort (**Figure 2A**). Additionally, the cAMP analog 8-Br-cAMP was also added to the sorted cultures to see the effects of PKA activation on ILC2 cells. Culturing our cells in the described conditions resulted in a greater than 90% ILC2 population identified as CD45^+^ lineage^-^CD25^+^ by the end of the incubation period (**Figure 2C**). In addition, the frequency of GATA-3 expressing cells was above 90% regardless the culture conditions tested (**Figure 2D**), confirming their identity as ILC2 cells.

Finally, we assessed the expression of CD127, another marker associated with the ILC family, in our cultures at different stages of our model. We found that throughout the protocol, the expression of this receptor is lost. In the newly isolated bone marrow all the CD25^+^ cells express CD127; however, after the initial 3-day incubation only 35% of the CD25^+^ ILC2 cells maintained the expression of this marker. By day 7 and depending on the treatment, only between 3 to 30% of CD25^+^ ILC2 cells expressed CD127 (**Supplementary Figure 2B**). For this reason, we decided to exclude CD127 as a selective marker for isolation of ILC2s in our model.

### IL-33 and cAMP Enhance IL-9 Expression and Regulate Specific Markers in Bone Marrow Derived ILC2 Cells

**Figure 3:**
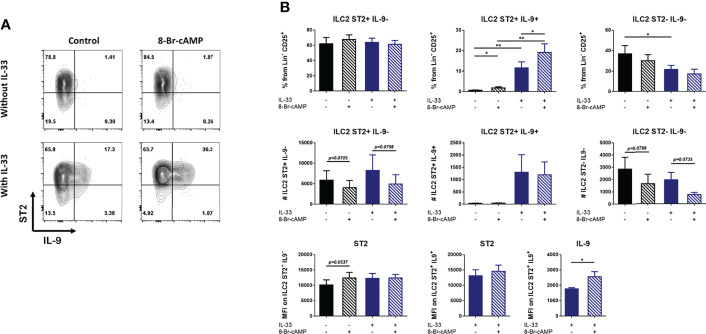
IL-33 and cAMP enhance IL-9 expression in BM-ILC2 cultures. **(A)** Representative dot plots of ILC2 populations generated with IL-2, IL-7, IL-25 and TSLP in the presence or absence of IL-33 and 8-Br-cAMP at day 7 of the BM-ILC2 culture (gated on live CD45^+^ lineage^-^ CD25^+^ cells). **(B)** Frequency, absolute numbers and MFI of ST2 and IL9 in ILC2 populations generated with IL-2, IL-7, IL-25 and TSLP in the presence or absence of IL-33 and 8-Br-cAMP at day 7 of the BM-ILC2 culture. Data represent the mean ± SEM of seven mice analyzed per group in 6 independent experiments. *p < 0.05, **p < 0.01.

The presence or absence of IL-33 in our BM-ILC2 cultures allowed us to identify three distinct populations based on the expression of ST2 and IL-9 (**Figure 3A**). No expression of IL-9 is observed in the absence of IL-33, therefore we were able to identify the ST2^+^ IL-9^-^ and ST2^-^IL-9^-^ ILC2 populations, while the addition of IL-33 resulted in the generation of an additional ILC2 population that expresses ST2 and IL-9 (**Figure 3A**); demonstrating that IL-33 is an important signal for IL-9 induction in our system.

While IL-33 decreased the number of ST2^+^ cells in whole bone marrow cultures (**Figure 2A**), no regulation of ST2 induced by IL-33 or PKA was observed in isolated ILC2s (**Figure 3A**). However, IL-33 treatment did increase the frequency and absolute numbers of IL-9-expressing ILC2 cells (**Figure 3B**). Treatment with 8-Br-cAMP also resulted in increased expression of IL-9 and the analog further synergized with IL-33 in the induction of ST2^+^ IL-9^+^ cells, increasing the MFI of IL-9 on these cells (**Figure 3B** lower panel). The positive regulation of IL-9 by IL-33 has been described in lung resident ILC2s (30), indicating that ILC2s induced by our model share this regulation with other tissue resident ILC2s.

We also evaluated the expression of CD25, KLRG1 and CD127, markers associated with ILC2s (**Supplementary Figure 3**). Our results indicate that IL-33 increases the expression of CD25 while we did not detect any type of PKA-mediated regulation of this marker (**Supplementary 3A, 3B**). In addition, the percentage of ILC2 expressing KLRG1 is increased by IL-33 but this increase is inhibited by PKA (**Supplementary Figures 3C, D**). Finally, IL-33 does not seem to induce changes in the expression of CD127; however, in the presence of this alarmin, activation of PKA leads to increased frequencies of ST2^+^ IL-9^-^ ILC2 cells expressing CD127 (**Supplementary Figures 3E, F**).

In conclusion, IL-33 and PKA activation synergize to induce IL-9 expression but differentially regulate other markers of ILC2 *in vitro*.

### cAMP Inhibits ILC2 Proliferation *In Vitro*

Despite the fact that we did not observe significant differences in the numbers of ILC2 cells associated with PKA activation, there is a trend indicating a possible decrease in the numbers of these cells in the presence of the cAMP analog (**Figure 3B**). With this data in mind and based on reports about PKA activity in T cells (11, 12), we decided to analyze whether PKA is capable of regulating cellular processes such as proliferation and apoptosis that could impact ILC2 numbers. To address this, we performed a dye dilution assay. ILC2s from bone marrow cultures were stained with the Tag-it Violet dye at day 3 of our protocol, previous to the ILC2 cell sorting. The cells were then cultured under the different conditions and dye dilution was monitored at day 7. The division index was calculated in order to quantify cell proliferation.

**Figure 4:**
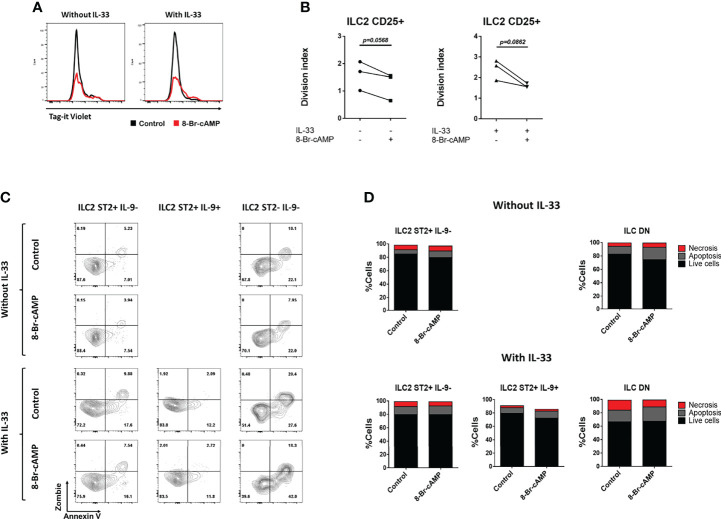
8-Br-cAMP regulates proliferation but not cell death of ILC2s. **(A)** Representative histograms of dye dilution in total ILC2s treated with or without 8-Br-cAMP in the presence or absence of IL-33 at day 7 of the BM-ILC2 culture (gated on live CD45^+^ lineage^-^ CD25^+^ cells). **(B)** Division index on total ILC2s treated with or without 8-Br-cAMP in the presence or absence of IL-33 at day 7 of the BM-ILC2 culture. Data represent three mice analyzed per group in 3 independent experiments. **(C)** Representative dot plots for the annexin V and viability staining (zombie) of the different ILC2 populations treated with or without 8-Br-cAMP in the presence or absence of IL-33 at day 7 of the BM-ILC2 culture. **(D)** Frequencies of living, apoptotic and necrotic cells of ILC2 populations generated in the presence (lower panel) or absence (upper panel) of IL-33 at day 7 of the BM-ILC2 culture. Data represent the mean of seven mice analyzed per group in 6 independent experiments.

Due to limited numbers of some ILC2 populations in our purified cultures, we could not evaluate the effect of 8-Br-cAMP on each different ILC2 subset; therefore, proliferation was assessed on the total CD45^+^ lineage^-^ CD25^+^ cells. The division index of cells with no IL-33 treatment was lower compared to the IL-33-treated culture, which is consistent with the capacity of IL-33 as a proliferative stimuli for ILC2 (**Figures 4A, B**). The cAMP analog induced an IL-33-independent proliferation arrest, as shown by the histograms and division index graphs in **Figures 4A, B**. These trending decreases are consistent in the 3 independent experiments performed. In conclusion, 8-Br-cAMP treatment can inhibit proliferation of our cells in an IL-33-independent manner, recapitulating one of the effects of the PKA activation previously described on T cells (11, 12).

### cAMP Does Not Regulate ILC2 Survival *In Vitro*

PKA can induce apoptosis of T lymphocytes (10), therefore we tested whether this signaling pathway works similarly on ILC2s by determining the percentages of living, apoptotic and necrotic cells in our ILC2 cultures in the presence or absence of IL-33 and 8-Br-cAMP (**Figure 4C**). We stained the ILC2s with a viability dye (Zombie) and Annexin V, which allows for the discrimination between viable cells (Annexin V^-^ Zombie^-^), cells in early apoptosis (Annexin V^+^ Zombie^-^) and cells in late apoptosis or necrosis (Annexin V^+^ Zombie^+^). The cellular status in our cultures was measured at day 7. No significant difference in the frequencies of live, apoptotic and dead cells was observed in any of the culture conditions analyzed (**Figures 4C, D**), leading us to conclude that under this model the PKA pathway does not regulate apoptosis of any of the ILC2 populations. However, our data suggest that expression of ST2 or IL-9 improves viability on ILC2 cells, as percentages of live cells are higher in ST2^+^IL-9^-^ and ST2^+^IL-9^+^ ILC2 cells compared with DN ILC2s cultured in the presence of IL-33, further supporting a positive signal promoted by IL-33 and IL-9 on ILC2s (18, 19).

### Characterization of the Cytokine Expression Profile in ILC2s in the Presence of cAMP *In Vitro*

ILC2s are an important source of type 2 cytokines in different inflammatory models (31–33). Several reports suggest that ligands like neuropeptides and lipid mediators that activate ILC2s and regulate the expression of IL-5 and IL-13, could also activate the PKA pathway (14, 20, 34). Hence, we analyzed the expression of type 2 cytokines and the effect of PKA activation on the different ILC2 populations obtained from our *in vitro* model. For this purpose, sorted ILC2s were cultured in the presence or absence of IL-33 with or without the cAMP analog to determine the effect of PKA activation on the induction of other type 2 cytokines. The different ILC2 populations were stained for IL-5 and IL-13 and analyzed by flow cytometry.

**Figure 5:**
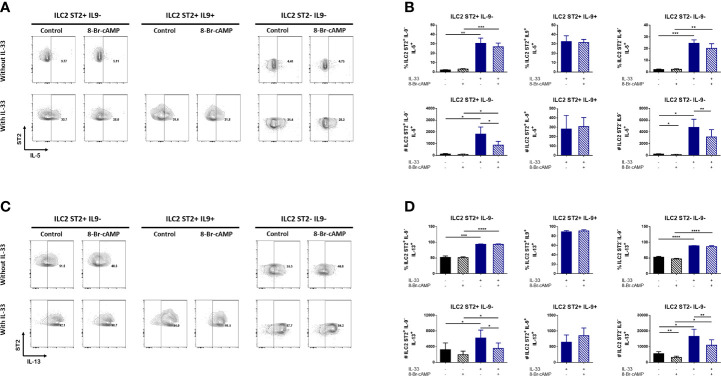
cAMP regulates IL-5 and IL-13 expression in IL-9 negative bone marrow derived ILC2s. **(A)** Representative dot plots of IL-5 expression in different ILC2 populations in the presence or absence of IL-33 and 8-Br-cAMP at day 7 of the BM-ILC2 culture. **(B)** Frequency and absolute numbers of IL-5 expression in different ILC2 populations shown in **(A)**. **(C)** Representative dot plots of IL-13 expression in different ILC2 populations in the presence or absence of IL-33 and 8-Br-cAMP at day 7 of the BM-ILC2 culture. **(D)** Frequency and absolute numbers of IL-13 expression in different ILC2 populations shown in **(C)**. Data represent the mean ± SEM of seven mice analyzed per group in 6 independent experiments. *p < 0.05, **p < 0.01, ***p < 0.001, ****p < 0.0001.

The percentages and numbers of cells expressing IL-5 significantly increased only in the presence of IL-33 (**Figures 5A, B**). In contrast, PKA activation resulted in decreased numbers of IL-5^+^ ILC2 cells; however, this inhibitory effect was restricted to ILC2s that do not express IL-9 (**Figures 5A, B**). Hence, IL-5 expression appears to be dependent on IL-33 while PKA activation negatively regulates it.

IL-33 was not required for IL-13 expression. Even in the absence of this alarmin, 50% of ILC2s are positive for IL-13, nevertheless we do observe significantly higher expression on IL-13 induced by IL-33 (**Figures 5C, D**). Similarly to the observed effects on IL-5, PKA activation decreases the numbers of IL-33-induced IL-13^+^ ILC2 cells that do not express IL-9 (**Figures 5C, D**). We also observed a particular effect of cAMP decreasing the numbers of ST2^-^IL-9^-^ ILC2 cells expressing IL-13 that was independent of IL-33 (**Figure 5D**).The MFI data in our ILC2 model shows a modest yet significant PKA-associated difference in IL-13 (**Supplementary Figure 4C**). Although the MFI of IL-13 increases significantly with IL-33, a decrease in IL-13 associated with PKA activation is observed in ST2^-^ IL-9^-^ ILC2 cells in the absence of IL-33 (**Supplementary Figure 4C**). This indicates that PKA can regulate certain ILC2 markers in the absence of this alarmin.

Since the discovery of Th9 cells as a specialized population different from Th2 cells, the expression of IL-4 and IL-9 in T lymphocytes was found to be mutually exclusive (17, 35). To determine if this occurs in a similar way in ILC2s, we used a KN2 reporter mouse in heterozygosis which expresses the human CD2 protein under the endogenous IL-4 promoter (26) to monitor the expression of IL-4. Different stimuli have been reported to induce the expression of IL-4 in ILC2 cells. However, we were unable to detect the presence of IL-4 in any of the culture conditions tested in this work (**Supplementary Figure 4D**). Thus, we conclude that IL-33 and cAMP are not sufficient stimuli to induce IL-4 in ILC2s *in vitro*.

Together, these results suggest that in the presence of IL-33, PKA activation have opposite effects regulating the numbers of IL-5 and IL-13 expressing cells but only in IL-9 negative ILC2s. A similar effect was observed in the MFI of IL-13 in ST2^+^IL-9^-^ and ST2^-^IL-9^-^ ILC2 cells. Finally we believe that other stimuli different from IL-33 and cAMP are necessary to induce IL-4 in ILC2 cells in our culture.

## Discussion

In this work we report the presence of distinct ILC2 populations based on ST2 and IL-9 expression in a helminth infection model. These populations, which include ST2^+^ IL-9^-^, ST2^+^ IL-9^+^, ST2^-^ IL-9^+^ and ST2^-^ IL-9^-^ had not been previously reported *in vivo*. Here, we provide the first evidence that they are found in tissues and to a lesser extent in lymphoid organs. The ST2^+^ IL-9^-^ cells predominate in the lung, and along with ST2^+^ IL-9^+^ cells, increase in the *N. brasiliensis* infection model. Infection with the parasite seems to induce the expression of ST2 and IL-9 in the intraepithelial compartment of the small intestine, however this induction is very discrete and did not reach the statistical significance. In the lamina propria, both IL-9^+^ populations are increased, however the ST2^-^ IL-9^+^ is the most abundant subset induced by the infection. Two different populations of ILC2 cells have been described: natural (nILC2), characterized by the expression of KLRG1 and ST2; and inflammatory (iILC2), which are induced by *N. brasilienis* infection and express KLRG1 but apparently lack ST2 (36, 37). This absence of ST2 expression on iILC2s might be explained by downregulation of the receptor driven by its ligand IL-33 in the small intestine, as recently demonstrated (38). IL-33 is constitutively expressed in this tissue and, together with IL-25, is further induced by *N. brasiliensis* infection to promote the generation of iILC2 cells. Hence, the presence of the ST2^-^ IL-9^+^ ILC2 population in the small intestine described here might be explained by this regulatory loop, where IL-33 and IL-25 are induced upon infection, promoting iILC2s that lack ST2 and express effector cytokines *in vivo*. Altogether, our results demonstrate a dynamic transformation of the ILC2 subsets dependent on the tissue and time of infection that can be modulated by the inflammatory context.

An important aspect to consider is that isolating cells from *N. brasiliensis* infected small intestine is extremely challenging and results in low frequencies of live cells, potentially masking real absolute numbers of cells present in the tissue *in vivo*. However, analyses of cell frequencies are feasible and reproducible using our protocol. To our knowledge, this is the first report showing hematopoietic cell isolation from *N. brasiliensis* infected small intestine, followed by analysis of ILC2 cells by flow cytometry, and hence represents a useful tool to evaluate these and other immune cells from this particular complicated tissue.

In order to dissect the signals that originate these ILC2 populations, we developed an *in vitro* culture model from bone marrow cells in which different combinations of cytokines were tested to determine which ones are important for the induction of ST2 and IL-9. We found that IL-33 is not necessary for the induction of its receptor since the combination of IL-2, IL-7, IL-25 and TSLP is sufficient to generate ST2^+^ ILC2s. On the contrary, IL-33 decreased ST2 expression, which could be due to internalization of the receptor upon ligand binding as described above. Additionally, our studies confirmed IL-33 as a key stimulus to increase IL-9 expression in our cells. This data agrees with previous reports by Mohapatra et al., where lung ILC2s expressed IL-9 in response to IL-33 and this expression was increased when the treatment was performed in combination with other cytokines such as IL-2, IL-7 and TSLP (30). Therefore, we can conclude that the ILC2s in the whole bone marrow culture model resemble the behavior of ILC2s derived from other tissues such as small intestine and lung.

In order to determine the effects of IL-33 and PKA activation directly on ILC2s, we developed another culture model termed BM-ILC2, in which we expanded bone marrow ILC2s in the presence of IL-2, IL-7, TSLP and IL-25 for 3 days. ILC2s where then sorted and cultured 4 additional days in the same cocktail with the addition of IL-33 and/or a cAMP analog. We further demonstrated that with our novel experimental strategy, we were able to generate cultures highly enriched on ILC2 cells expressing GATA-3 and CD25, as expected.

In this model, IL-33 did not have a negative effect on the expression of ST2, differing from the results observed in the whole bone marrow culture. We believe that the effect observed in total bone marrow cultures may depend on further signals provided by other cells, in addition to the presence of IL-33. On the other hand, the IL-33-dependent induction of IL-9 on our BM-ILC2 cells was independent of those potential signals included in the total bone marrow culture, since purified cultures of ILC2s exposed to IL-33 still showed a strong induction of IL-9.

The addition of IL-33 to our sorted ILC2 cultures increased the expression of other markers associated with the identification of these cells such as CD25 and KLRG1. In addition, the differential expression of IL-9 generated three different subpopulations of ILC2 cells, all identified as CD45^+^ lineage^-^ CD25^+^ GATA-3^+^. These subsets are ST2^+^ IL-9^-^, ST2^+^ IL-9^+^ and ST2^-^ IL-9^-^. The first 2 populations correspond to those previously found in the lung and in the intraepithelial compartment of the small intestine. The ST2^-^ IL-9^+^ ILC2 population identified in lamina propria of *N. brasiliensis* infected mice, could not be induced in our *in vitro* model; we found the frequencies of these cells to be less than 3 percent of the total population. As previously mentioned this could be due to the requirement of other signals in combination with IL-33 to induce the negative regulation of ST2 while maintaining the expression of IL-9.

The PKA pathway is known to regulate the expression of cytokines in T lymphocytes and ILC2s. Here, we confirmed that 8-Br-cAMP-mediated PKA activation in the presence of IL-33 increased IL-9 expression, which does not modify the distribution of the ILC2 populations but could enhance the function of ST2^+^ IL-9^+^ ILC2 cells. To our knowledge, there are no previous reports associating PKA activation with an increase in IL-9 in innate lymphoid cells, however this effect has been observed in Th9 lymphocytes where the CGRP/cAMP/PKA pathway promotes IL-9 production *via* NFATc2 activation (39). This establishes a possible common regulatory pathway between ILC2 and T lymphocytes.

The synergic effect of PKA and IL-33 on IL-9 expression described here is contrasting with studies that report PKA-mediated inhibition of type 2 cytokines in ILC2s (14, 20, 34). This suggests that although our *in vitro* ILC2s have characteristics in common with lung ILC2s, there are also specific differences associated with the microenvironment of these cells. Along with the increase in IL-9, PKA activation induced CD127 and decreased KLRG1 expression. Previous reports have identified KLRG1 not only as a marker of ILC2 maturation but also as a negative regulator of the function of these cells. The interaction of activated ILC2s with the KLRG1 ligand E-cadherin results in a down regulation of GATA-3, IL-5, IL-13, amphiregulin and reduced ILC2 proliferation (29). In other tissues such as the stomach, ILC2s can be activated by IL-7, inducing proliferation and expression of cytokines (40). We propose that PKA activation could prime these cells for a better activated phenotype, perhaps as a mechanism to ensure proliferation and proper function. However, future experiments are necessary to verify this.

A striking finding is that when PKA is activated, ILC2s seem to decrease in numbers except for the IL-9-producing population. Our results suggest that this can be attributed to the inhibition of proliferation since we did not find PKA activation to be associated with apoptosis. However, the induction of IL-9 by PKA could be compensating for these proliferation effects, which is why we do not see differences in absolute numbers of IL-9^+^ ILC2 cells when we activate PKA. Our results are in accordance with previous studies that report IL-9 to be a potent inducer of proliferation and survival for ILC2 cells (19).

The inhibitory effects on proliferation described here for ILC2s have already been reported in T cells (11, 12, 41). In this model, inhibition of proliferation by the cAMP analog is observed independently of IL-33. cAMP has been reported to inhibit proliferation in T cells through different mechanisms. For example, it can generate cell cycle arrest through the inhibition of cyclins D and E (12) and PKA activation can block IL-2 receptor signaling (11). Further research is necessary to determine the regulatory mechanism carried out by PKA to inhibit proliferation of the ILC2s in our model. In addition, although we did not observed an effect on apoptosis induced by IL-33 and the PKA pathway in our cultures, we did see a lower frequency of apoptotic and death cells on the ILC2 subset expressing IL-9, further supporting the role of IL-9 as a positive signal acting in an autocrine way on these cells (18, 19).

Additional data derived from our BM-ILC2 cultures validate this protocol as a means to generate bona fide ILC2 cells, since our ILC2 populations express both IL-5 and IL-13. In agreement with other works, our data indicate that IL-5 induction is dependent on IL-33 as has been observed in lung-derived ILC2s (42). Contrastingly, IL-13 expression does not require IL-33, however it is significantly enhanced by the presence of the alarmin. Our data is in agreement with previous reports of ILC2s in the intestine, where IL-25 produced by Tuft cells further activated these cells to produce IL-13 in a parasitic infection context (43).

Lung ILC2s have been reported to express IL-5 and IL-13, which can be blocked by cAMP analogs and ligands that activate PKA (13). The effects of PKA on these two cytokines are similar in our model. We established that cAMP has regulatory effects on the numbers of ILC2 cells expressing IL-5 and/or IL-13. These effects were observed only in the presence of IL-33 and were restricted to ILC2s that do not express IL-9. These data suggest that while there is a decrease in the induction of IL-5 and IL-13 in ILC2 cells, the increase in IL-9-expressing cells compensates for these defects, resulting in no change in the absolute numbers of ILC2 that co-express IL-5 or IL-13 with IL-9.

Finally, the induction of IL-4 has been reported in ILC2s through activation with other mediators such as leukotriene D4, but not with IL-33 (44, 45); further supporting the observation in our model that IL-33 alone is unable to promote IL-4 expression *in vitro*. This could be because in addition to the cytokines used here and in previous reports, other signals are required to generate IL-4^+^ ILC2 cells; however, this has yet to be demonstrated.

In general, the ILC2s differentiated from bone marrow precursors obtained with our *in vitro* model share various characteristics with tissue-derived ILC2s. It is tempting to speculate that the different ILC2 subpopulations characterized in this work are found in different anatomical locations and that their modulation depends on the conditions and time of stimuli presentation. *N. brasiliensis* infection for example, results in tissue damage and IL-33 release (46). Worm-derived factors can modulate this IL-33 response (25) and consequently generate the different ILC2 populations. Therefore, it would be interesting to determine if these ILC2 populations are induced in response to IL-33 *in vivo* as well. Here, we found that stimulation with IL-33 and PKA activation results in an enrichment of IL-9^+^ ILC2 cells that are competent regarding the expression of IL-5 and IL-13. Therefore, expansion of this population could contribute to immunity against helminth infections.

Based in our results, the PKA pathway antagonizes IL-33 in several processes such as proliferation and production of IL-5 and IL-13, while inducing a phenotype that favors its ability to respond to other stimuli in the microenvironment (IL-9^+^, CD127^+^, KLRG1^-^). Whether changes in PKA activation are generated during helminth infection with *N. brasiliensis* is a matter of interest and remains to be determined along with the identification of the potential ligands promoting such activation *in vivo*.

To conclude, the data presented here provide further evidence of the high heterogeneity between ILC2 subsets in a helminth infection context and calls for careful consideration when choosing the markers to identify these cells. In addition, by providing a protocol for differentiation and expansion of bona fide ILC2 cells, we contribute with a useful tool for studies focused on ILC2 biology. Finally, the findings that IL-33 and PKA induce IL-9 and regulate the different ILC2 population dynamic, could help us better understand the physiology of these cells, allowing their manipulation for therapeutic purposes in specific anatomical locations.

## Data Availability Statement

The raw data supporting the conclusions of this article will be made available by the authors, without undue reservation.

## Ethics Statement

The animal study was reviewed and approved by CICUAL Instituto de Fisiología Celular, UNAM.

## Author Contributions

EO-M conceived, design and performed the experiments, analyze the data and wrote the manuscript, OM-P performed experiments and discussed the data, BER-M revised the manuscript, JLR-B performed experiments, acquired the data and provided reagents, IL-L discussed and thoroughly revised the results and conclusions of the manuscript, PL-L conceived the idea, design the experiments, discussed and revised the manuscript, supervised the project and acquired the funding. All authors contributed to the article and approved the submitted version.

## Funding

This work was supported by the following grants to PLL from CONACYT (CB 255287 FORDECYT-PRONACE-303027) and DGAPA (IN209919-PAPIIT). EO-M received a fellowship from CONACYT (481437).

## Conflict of Interest

The authors declare that the research was conducted in the absence of any commercial or financial relationships that could be construed as a potential conflict of interest.

## Publisher’s Note

All claims expressed in this article are solely those of the authors and do not necessarily represent those of their affiliated organizations, or those of the publisher, the editors and the reviewers. Any product that may be evaluated in this article, or claim that may be made by its manufacturer, is not guaranteed or endorsed by the publisher.

## Supplementary Material

Supplementary Figure 1 INFER mice were subcutaneously infected with 200 N*. brasiliensis* larvae subcutaneously and ILC2s from lung and small intestine draining lymph nodes were analyzed at different times post-infection. **(A)** Gating strategy to identify the different ILC2 populations in tissues. **(B)** Absolute numbers of the different ILC2 subsets in the lung on day 0 (control), 4, 7 and 10 post-infection. **(C)** Representative dot plots of different ILC2 populations generated in the mediastinal lymph nodes on day 0 (control) and 10 post-infection (gated on live CD45^+^ lineage^-^ CD90^+^ cells). **(D)** Frequencies and absolute numbers of the different ILC2 subsets in the mediastinal lymph nodes on day 0 (control), 4, 7 and 10 post-infection. **(E)** Representative dot plots of different ILC2 populations generated in the mesenteric lymph nodes on day 0 (control), and 10 post-infection (gated on live CD45^+^ lineage^-^ CD90^+^ cells). **(F)** Frequencies and absolute numbers of the different ILC2 subsets in the mesenteric lymph nodes on day 0 (control), 4, 7 and 10 post-infection. Data represent the results of two (days 4 and 10) or four independent experiments (day 7) with one or two mice analyzed per dot. *p<0.05, **p<0.01, ***p<0.001, , ****p<0.0001

Supplementary Figure 2 **(A)** Gating strategy used for sorting ILC2s at day 3 of the BM-ILC2 culture and purity post-sort of those cells. **(B)** Representative dot plots of CD127 expression on different days of BM-ILC2 culture (gated on CD45^+^ lineage-CD90^+^ for the days 0 and 3 and CD45^+^ lineage- for the day 7). **(C)** Representative fluorescence minus one (FMO) dot plots for the markers used in the sort and analysis of ILC2s, controls were performed in total bone marrow cultures to ensure positive and negative populations.

Supplementary Figure 3 **(A)** Representative histograms of CD25 expression in different ILC2 populations in the presence or absence of IL-33 and 8-Br-cAMP at day 7 of the BM-ILC2 culture. **(B)** MFI of CD25 expression in different ILC2 populations shown in **(A)**. **(C)** Representative dot plots of KLRG1 expression in different ILC2 populations in the presence or absence of IL-33 and 8-Br-cAMP at day 7 of the BM-ILC2 culture. **(D)** Frequency of KLRG1 expression in different ILC2 populations shown in **(A)**. **(E)** Representative dot plots of CD127 expression in different ILC2 populations in the presence or absence of IL-33 and 8-Br-cAMP at day 7 of the BM-ILC2 culture. **(F)** Frequency of CD127 expression in different ILC2 populations shown in **(A)**. Data represent the mean ± SEM of 7, 6 or 5 mice analyzed per group for CD25, KLRG1 and CD127 respectively. *p<0.05, **p<0.01, ***p<0.001.

Supplementary Figure 4 **(A)** Representative FMO dot plots for IL-5 and IL-13 staining. **(B)** MFI of IL-5 expression in different ILC2 populations with or without 8-Br-cAMP in the presence of IL-33 at day 7 of the BM-ILC2 culture. **(C)** MFI of IL-13 expression in different ILC2 populations in the presence or absence of IL-33 and 8-Br-cAMP at day 7 of the BM-ILC2 culture. **(D)** Representative dot plots of IL-4 expression on total ILC2s from a KN2 mice, in the presence or absence of IL-33 and 8-Br-cAMP at day 7 of the BM-ILC2 culture. Data represent the mean ± SEM of seven mice analyzed per group in 6 independent experiments. *p<0.05, **p<0.01, ****p<0.0001.
