# Supplementary figures and images for: IL-33 and the PKA Pathway Regulate ILC2 Populations Expressing IL-9 and ST2
Source: Front Immunol. 2022 May 30;13:787713. doi: 10.3389/fimmu.2022.787713 (PMC9197159; doi:10.3389/fimmu.2022.787713)

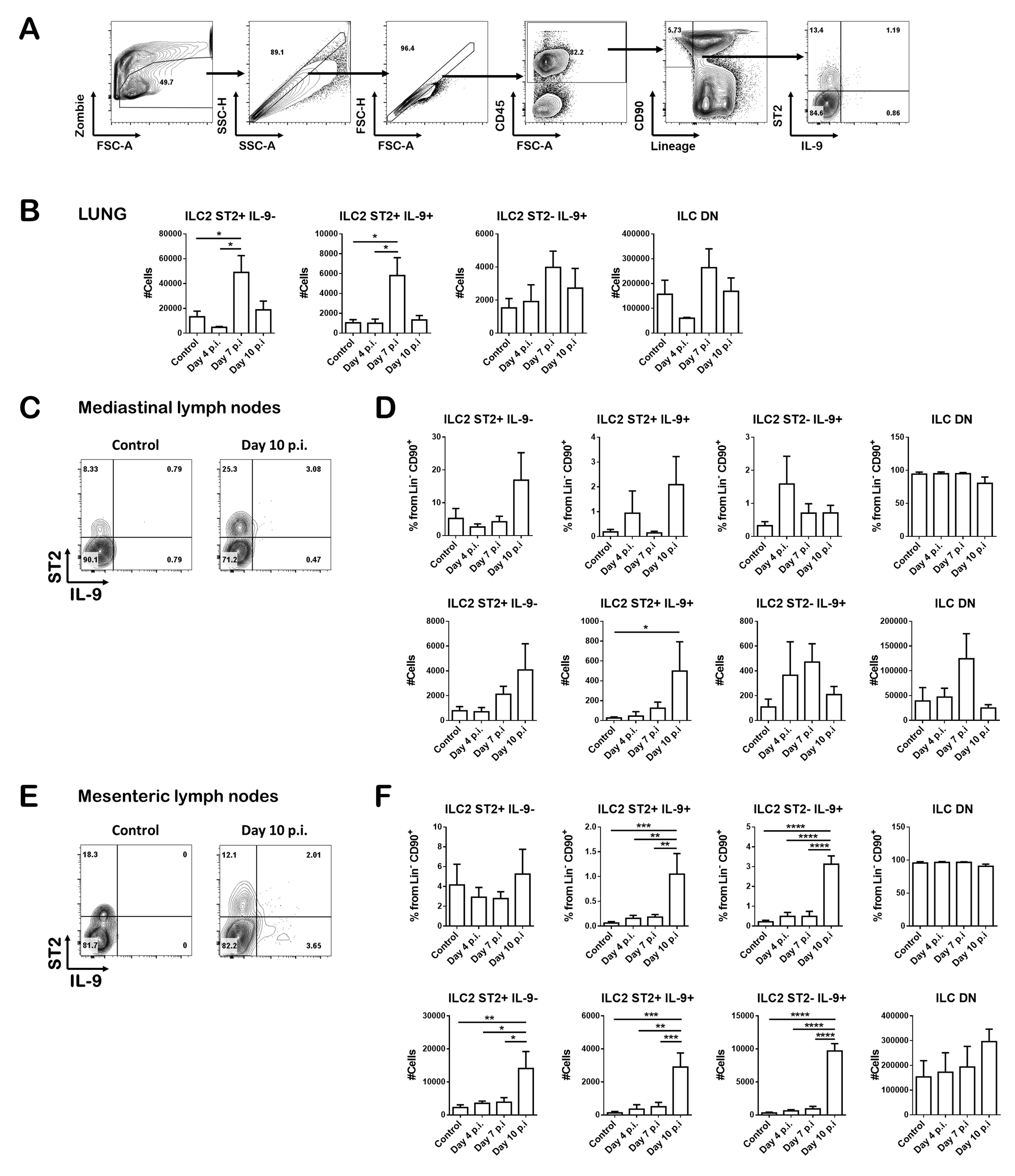

Supplement: Supplementary Figure 1 — INFER mice were subcutaneously infected with 200 N. brasiliensis larvae subcutaneously and ILC2s from lung and small intestine draining lymph nodes were analyzed at different times post-infection. (A) Gating strategy to identify the different ILC2 populations in tissues. (B) Absolute numbers of the different ILC2 subsets in the lung on day 0 (control), 4, 7 and 10 post-infection. (C) Representative dot plots of different ILC2 populations generated in the mediastinal lymph nodes on day 0 (control) and 10 post-infection (gated on live CD45+ lineage- CD90+ cells). (D) Frequencies and absolute numbers of the different ILC2 subsets in the mediastinal lymph nodes on day 0 (control), 4, 7 and 10 post-infection. (E) Representative dot plots of different ILC2 populations generated in the mesenteric lymph nodes on day 0 (control), and 10 post-infection (gated on live CD45+ lineage- CD90+ cells). (F) Frequencies and absolute numbers of the different ILC2 subsets in the mesenteric lymph nodes on day 0 (control), 4, 7 and 10 post-infection. Data represent the results of two (days 4 and 10) or four independent experiments (day 7) with one or two mice analyzed per dot. *p<0.05, **p<0.01, ***p<0.001, , ****p<0.0001 [file Image_1.tiff]

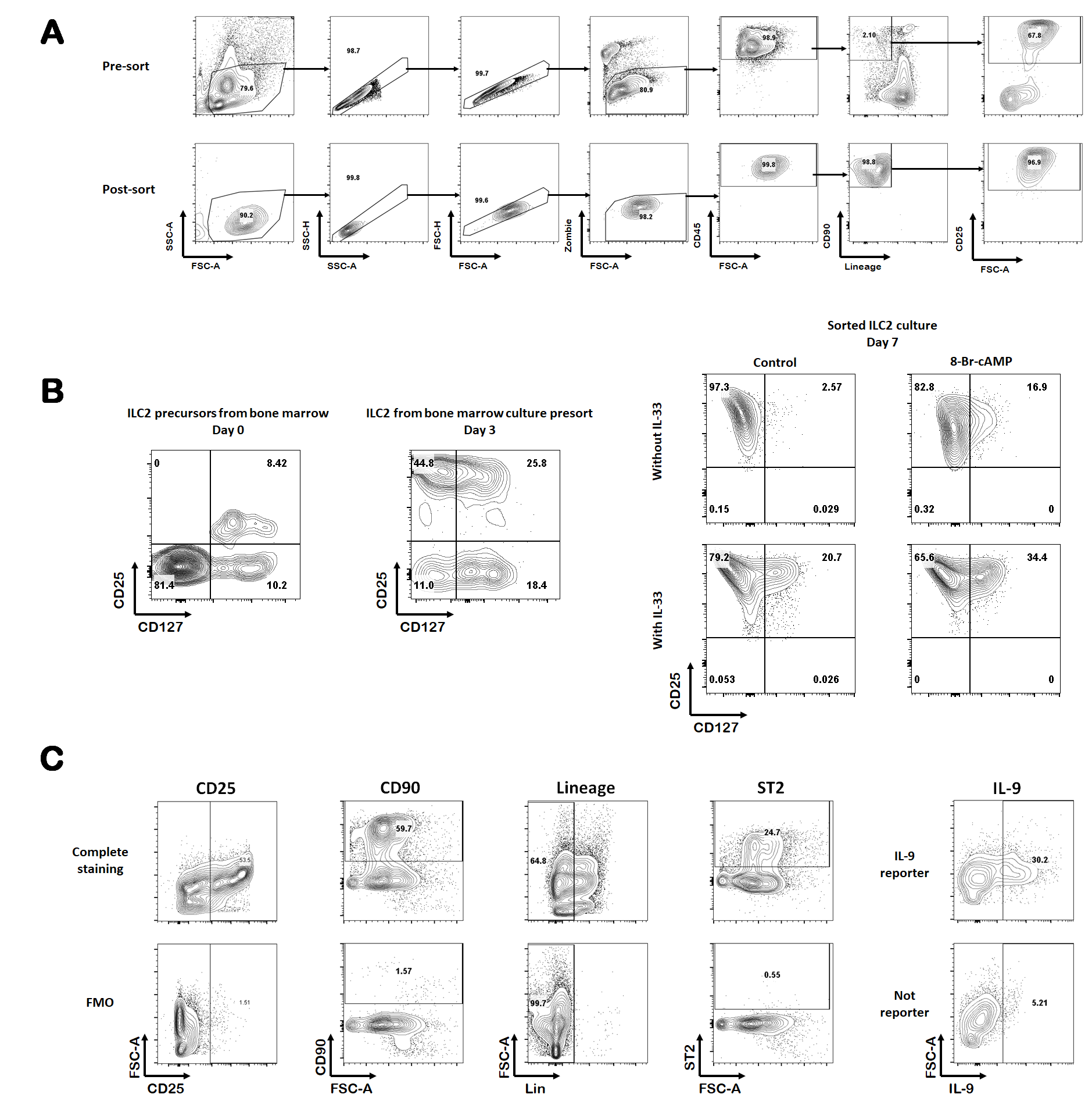

Supplement: Supplementary Figure 2 — (A) Gating strategy used for sorting ILC2s at day 3 of the BM-ILC2 culture and purity post-sort of those cells. (B) Representative dot plots of CD127 expression on different days of BM-ILC2 culture (gated on CD45+ lineage-CD90+ for the days 0 and 3 and CD45+ lineage- for the day 7). (C) Representative fluorescence minus one (FMO) dot plots for the markers used in the sort and analysis of ILC2s, controls were performed in total bone marrow cultures to ensure positive and negative populations. [file Image_2.tiff]

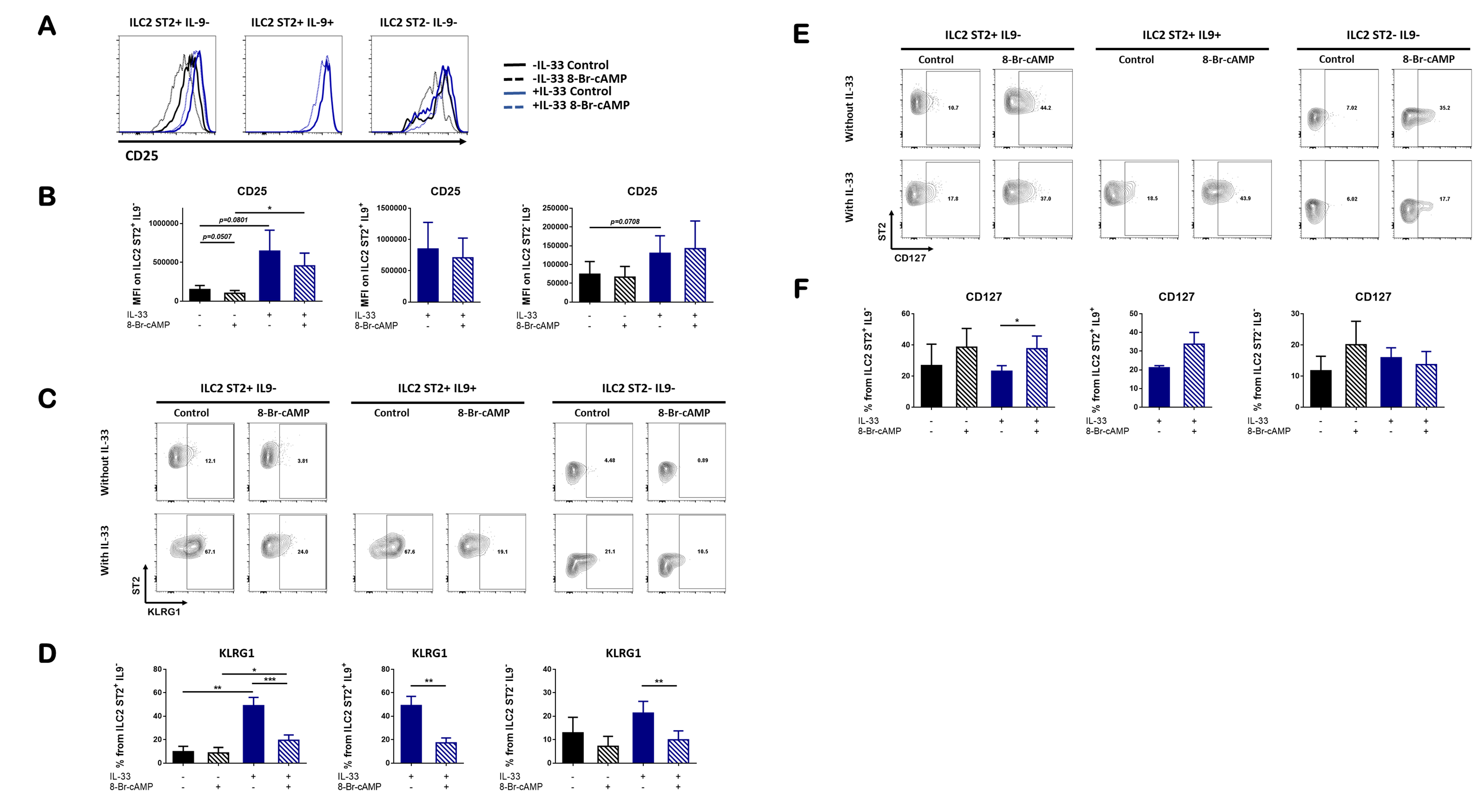

Supplement: Supplementary Figure 3 — (A) Representative histograms of CD25 expression in different ILC2 populations in the presence or absence of IL-33 and 8-Br-cAMP at day 7 of the BM-ILC2 culture. (B) MFI of CD25 expression in different ILC2 populations shown in (A). (C) Representative dot plots of KLRG1 expression in different ILC2 populations in the presence or absence of IL-33 and 8-Br-cAMP at day 7 of the BM-ILC2 culture. (D) Frequency of KLRG1 expression in different ILC2 populations shown in (A). (E) Representative dot plots of CD127 expression in different ILC2 populations in the presence or absence of IL-33 and 8-Br-cAMP at day 7 of the BM-ILC2 culture. (F) Frequency of CD127 expression in different ILC2 populations shown in (A). Data represent the mean ± SEM of 7, 6 or 5 mice analyzed per group for CD25, KLRG1 and CD127 respectively. *p<0.05, **p<0.01, ***p<0.001. [file Image_3.tiff]

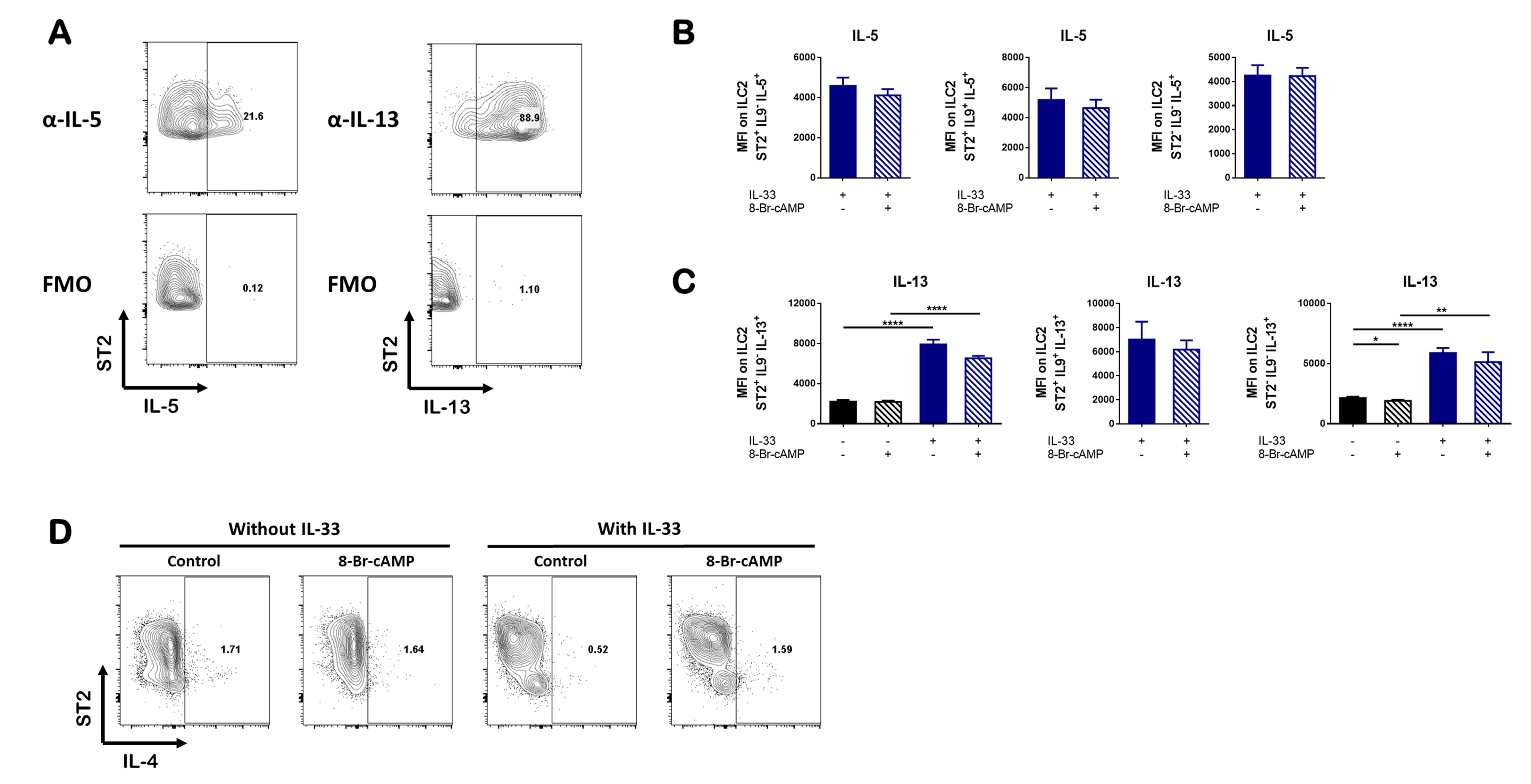

Supplement: Supplementary Figure 4 — (A) Representative FMO dot plots for IL-5 and IL-13 staining. (B) MFI of IL-5 expression in different ILC2 populations with or without 8-Br-cAMP in the presence of IL-33 at day 7 of the BM-ILC2 culture. (C) MFI of IL-13 expression in different ILC2 populations in the presence or absence of IL-33 and 8-Br-cAMP at day 7 of the BM-ILC2 culture. (D) Representative dot plots of IL-4 expression on total ILC2s from a KN2 mice, in the presence or absence of IL-33 and 8-Br-cAMP at day 7 of the BM-ILC2 culture. Data represent the mean ± SEM of seven mice analyzed per group in 6 independent experiments. *p<0.05, **p<0.01, ****p<0.0001. [file Image_4.tiff]
